# Automatic muscle impedance and nerve analyzer (AMINA) as a novel approach for classifying bioimpedance signals in intraoperative pelvic neuromonitoring

**DOI:** 10.1038/s41598-023-50504-7

**Published:** 2024-01-05

**Authors:** Ramona Schuler, Andreas Langer, Christoph Marquardt, Georgi Kalev, Maximilian Meisinger, Julia Bandura, Thomas Schiedeck, Matthias Goos, Albert Vette, Marko Konschake

**Affiliations:** 1 Research and Development, Dr. Langer Medical GmbH, Waldkirch, Germany; 2 Institute of Biomedical Engineering and Informatics, TU Ilmenau, Ilmenau, Germany; 3 Dr. Langer Consulting GbR, Langefurt 12, Waldkirch, Germany; 4 Department of General, Visceral, Thoracic and Pediatric Surgery, Ludwigsburg Hospital, Ludwigsburg, Germany; 5 Department of General and Visceral Surgery, Helios Klinik Müllheim, Müllheim, Germany; 6 Department of Mechanical Engineering, University of Alberta, Edmonton, AB T6G 1H9 Canada; 7 Glenrose Rehabilitation Hospital, Alberta Health Services, Edmonton, AB T5G 0B7 Canada; 8 Department of Anatomy, Histology and Embryology, Institute of Clinical and Functional Anatomy, Medical University of Innsbruck (MUI), Müllerstr. 59, 6020 Innsbruck, Austria

**Keywords:** Autonomic nervous system, Translational research, Electrical and electronic engineering

## Abstract

Frequent complications arising from low anterior resections include urinary and fecal incontinence, as well as sexual disorders, which are commonly associated with damage to the pelvic autonomic nerves during surgery. To assist the surgeon in preserving pelvic autonomic nerves, a novel approach for intraoperative pelvic neuromonitoring was investigated that is based on impedance measurements of the innervated organs. The objective of this work was to develop an algorithm called AMINA to classify the bioimpedance signals, with the goal of facilitating signal interpretation for the surgeon. Thirty patients included in a clinical investigation underwent nerve-preserving robotic rectal surgery using intraoperative pelvic neuromonitoring. Contraction of the urinary bladder and/or rectum, triggered by direct stimulation of the innervating nerves, resulted in a change in tissue impedance signal, allowing the nerves to be identified and preserved. Impedance signal characteristics in the time domain and the time–frequency domain were calculated and classified to develop the AMINA. Stimulation-induced positive impedance changes were statistically significantly different from negative stimulation responses by the percent amplitude of impedance change A_max_ in the time domain. Positive impedance changes and artifacts were distinguished by classifying wavelet scales resulting from peak detection in the continuous wavelet transform scalogram, which allowed implementation of a decision tree underlying the AMINA. The sensitivity of the software-based signal evaluation by the AMINA was 96.3%, whereas its specificity was 91.2%. This approach streamlines and automates the interpretation of impedance signals during intraoperative pelvic neuromonitoring.

## Introduction

Intraoperative pelvic neuromonitoring in visceral surgery provides surgeons an advantage in identifying pelvic autonomic nerves, which is crucial for the patient’s functional outcomes and quality of life. Intraoperative preservation of pelvic nerves can be challenging due to adverse anatomical situations, the fine and fragile phenotype of pelvic autonomic nerves, and their similarity to fat tissue, connective tissue, or scar tissue^1,2^. Recent studies report high rates of postoperative dysfunction of the innervated pelvic organs, with up to 38% of patients suffering from urinary incontinence and up to 46% of patients experiencing bladder voiding difficulties during a one to two year follow up period after surgical rectal cancer treatment^3–5^. Prevalence of the low anterior resection syndrome (LARS) varies in the range of 30% to 80%. The most commonly reported symptoms of LARS are incontinence, urgency, or outlet obstructions^6–8^. Besides preoperative radiation or traction of the nerves, operation-specific nerve damage is a critical risk factor for postoperative complications of this kind^5,7–10^, which highlights the importance of technical assistance in intraoperative pelvic nerve preservation.

The physical principle underlying intraoperative nerve identification in the context of intraoperative neuromonitoring involves direct stimulation of nerves in the surgical field with a hand held probe and electrophysiological recording of the targeted organ’s response^11^. Currently, the only commercially available approach to intraoperative pelvic neuromonitoring was developed by Kauff and Kneist et al.^12^. This method is based on direct pelvic nerve stimulation in the surgical field, electromyography (EMG) on the internal anal sphincter (IAS), and bladder manometry^13^. An increased EMG activity of the IAS in the frequency range of 5–20 Hz compared to the resting tonus of the muscle during direct stimulation of functional nerves is rated as a positive stimulation response. A positive bladder response is defined as an increased intravesical pressure caused by stimulation-induced bladder contraction, which requires bladder filling with Ringer’s solution for each repeated stimulation period^13–16^. With this method, the intraoperative workflow is repeatedly interrupted due to the time-consuming bladder filling required when using bladder manometry.

**Figure 1:**
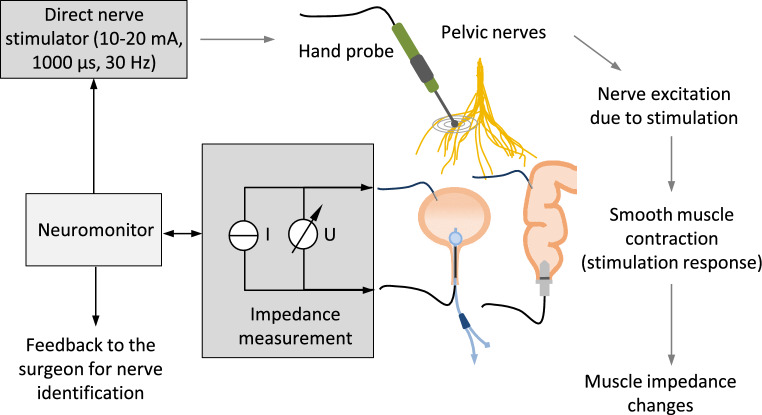
Pelvic neuromonitoring principle based on direct nerve stimulation with a hand probe and bioimpedance measurement of the bladder and rectum with a bipolar electrode montage. Contraction of the bladder and rectum smooth muscle in response to direct stimulation of the innervating autonomic nerves results in a change in the muscle tissue impedance compared to the impedance level before contraction. Assessment of stimulation-induced tissue impedance changes allows the nerves to be identified and preserved by the surgeon.

The research presented in this study focused on a novel approach to intraoperative pelvic neuromonitoring. This approach involved measuring bioimpedance on the urinary bladder and rectum, during direct nerve stimulation in the surgical field. The technical and clinical feasibility of this new method was demonstrated through two phases: a preclinical animal study involving twelve pigs followed by a clinical investigation that included 30 patients undergoing robotic rectal resections^17^. The method relies on the fundamental physical principle of inducing contraction in the smooth muscles of the bladder and rectum through direct stimulation of the innervating autonomic nerves, resulting in a change in the muscle tissue impedance compared to the impedance level before contraction. Assessment of stimulation-induced tissue impedance changes allows the surgeon to identify and preserve the nerves, a principle that is illustrated in Fig. 1. The method overcomes the disadvantage of repeated bladder filling as it is applicable to the empty bladder.

It was found that a change in the tissue impedance signal caused by a slow smooth muscle contraction is characterized by a specific signal shape. Other changes in the impedance signal caused by surgical manipulations, organ movements, or mechanical ventilation of the patient (i.e., artifacts) differ in signal morphology and frequency, but may complicate signal interpretation for the surgeon.

To enhance the efficiency of the surgeon’s workflow, the investigation of implementing an algorithm called the “Automatic muscle impedance and nerve analyzer” (AMINA) into a neuromonitoring device was conducted. This algorithm enables automatic evaluation of signals, aiming to streamline the surgical process. The goal of developing the AMINA was to automatically assess the response of the urinary bladder and rectum to stimulation of tissue in the surgical field, which may contain pelvic autonomic nerves. This should allow the surgeon to be aware of whether functional pelvic autonomic nerves are present in the area of the stimulation position, assisting or even replacing manual signal interpretation by an application specialist.

For this purpose, the AMINA should distinguish between the following three signal classes: (1) a positive stimulation-induced characteristic impedance change; (2) a negative stimulation response (no significant impedance change); and (3) a non-specific signal change (artifact). The objective of the present study is to introduce the AMINA based on discretized continuous wavelet transform (CWT) and demonstrate its validity, with the goal of automating smooth muscle impedance interpretation in intraoperative pelvic neuromonitoring.

## Methods

The clinical feasibility of bioimpedance measurement as a new pelvic neuromonitoring method was investigated in a clinical study. Measurement data utilized for the investigation, development, and subsequent testing of the AMINA, were collected within the confines of this study.

### Study design: clinical investigation

The study was a prospective clinical investigation that included a total of 30 patients undergoing surgical procedures such as rectal resection for rectal cancer or diverticulitis, resection rectopexy for rectal prolapse, or rectal extirpation for rectal cancer. Each patient underwent a preoperative assessment of urinary and fecal function, intraoperative application of pelvic neuromonitoring, and assessment of urinary and fecal function up to one year postoperatively. The study was approved by the Ethics Committee of the Landesärztekammer Baden-Württemberg (Application No. 00011915/00054594) and the Higher Federal Authority (BfArM) (Application No. 94.1.12-5660-11914). It is registered in the German Clinical Trials Register DRKS00017437, accredited by the German Cancer Society (DKG) Registry (No. ST-D528) and conducted according to the Declaration of Helsinki and the DIN EN ISO 14155 at Ludwigsburg Hospital. Informed consent was obtained from all participants or their legal guardians.

### Intraoperative neuromonitoring application

Intraoperative neuromonitoring was performed by direct nerve stimulation in the surgical field (direct nerve stimulation with monophasic square-wave pulses in the range of 10–20 mA, 1000 μs pulse width, and a pulse frequency of 30 Hz) using a handheld probe, and impedance measurement of the urinary bladder and rectum (see Fig. 1 for a schematic illustration). A nerve stimulator was used because the absolute current delivered remains unchanged over time, whereas the voltage per pulse is adjusted depending on the electrical quality of the electrode-tissue-interface. For impedance measurement on the two target organs (the bladder and rectum), two electrodes on each organ were applied, detecting the contractile tissue between the electrodes. A needle electrode (monopolar needle electrode, needle length 18 mm, needle diameter 0.4 mm, item no. MN4018D25S, Spes Medica S.r.l, Italy) was inserted at the bladder’s apex from the surgical site, and a catheter electrode (Disposable Urethral Catheter Electrode, size 14 Fr or 16 Fr, item no. UE002 or UE003, Spes Medica S.r.l, Italy) was positioned on the urethral sphincter. Thus, the tissue of the detrusor muscle between the needle electrode and urethral electrode is captured for impedance measurement. At the rectum, a needle electrode (monopolar needle electrode, needle length 18 mm, needle diameter 0.4 mm, item no. MN4018D25S, Spes Medica S.r.l, Italy) was used in the upper rectum and a rectal probe (Probe Electrode with ring electrodes, electrode surface width 10 mm for males, item no. 61013A; electrode surface width 5 mm for females, item no. 61020A, Everyway Medical Instruments Co., Ltd.) was positioned in the anal canal. The prototype of a new neuromonitoring system for pelvic autonomic nerve monitoring AVALANCHE® NeuroNeB (Dr. Langer Medical GmbH, Waldkirch, Germany) was used. Impedance measurement was carried out with a bipolar electrode montage using a testing current of 50 µA and 50 kHz. The voltage drop across the tissue was measured with a differential amplifier, amplified (gain of 50), high-pass filtered (cutoff frequency of 160 Hz), lock-in amplified using the testing current as reference signal, and analog-to-digital converted. Because of the capacitive behavior of biological tissue, there’s a phase shift between the measurement signal and the reference signal. Lock-in amplification was implemented via an alternating input of the sine reference signal and a 90°-phase-shifted second reference signal (a cosine reference signal) to the lock-in amplifier. This is followed by alternating multiplication of the measurement signal with the sine and the cosine reference signal, resulting in two output signal components. Demodulation (signal integration) was realized via a 3rd-order low-pass filter (lowest cutoff frequency of 5 Hz), resulting in a direct current (DC) signal. The DC signal is amplified (gain of 11) and fed into an analog–digital converter (ADC). ADC resolution was 14 bits at ± 10 V, and the sampling rate was set to 10 Hz. The DC signal represents a processed voltage drop U(t) proportional to the target organs’ tissue impedance, which was displayed on the neuromonitor as a function of time. The introduction of impedance measurement as a new technology in intraoperative pelvic neuromonitoring including hardware design and analog filtering was published in 2022^17^.

The application software of the neuromonitoring system stores the recorded data (processed voltage drop U(t)) in TDMS files (Technical Data Management Streaming, file format by National Instruments Corporation, Austin, USA) with a sampling rate of 10 Hz, which represent the input data for the algorithm development. No additional online signal processing was applied.

### Intraoperative signal pre-classification

**Figure 2:**
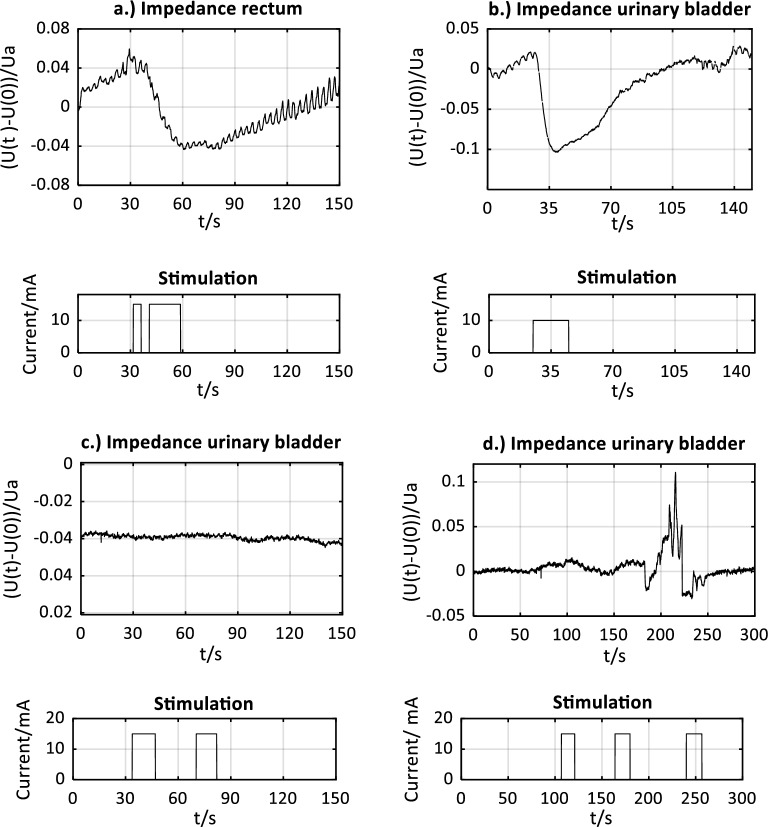
Signal examples of pre-classified signals of: (1) a positive stimulation-induced characteristic impedance change of the rectum (upper left, **a**), from case 7 of the clinical investigation/patient LB-09) and the urinary bladder (upper right, **b**), from case 1 of the clinical investigation/patient LB-01). The voltage drop (U(t)–U(0))/U_a_ across the smooth muscle changes during stimulation of autonomic nerves in the surgical field until the maximum impedance change is reached. This is followed by a relaxation phase of the muscle with the decrease of the voltage drop to the initial level. U(t) is the voltage drop as a function of time, U(0) is the voltage drop at time t = 0s, and U_a_ is the impedance level at baseline (mean value of the first 15 samples acquired within 1.5 s). Signal examples of pre-classified signals of: (2) a negative stimulation response (lower left, c.), from case 26 of the clinical investigation/patient LB-29; and (3) a non-specific artifact (lower right, **d**), from case 2 of the clinical investigation/patient LB-02).

When neuromonitoring was applied intraoperatively, an application specialist classified the displayed voltage drop U(t) across the target organs’ tissue and documented the result to identify whether a stimulation-induced impedance change occurred at the urinary bladder and/or rectum. Signal examples of pre-classified signals of: (1) a positive stimulation-induced characteristic impedance change; (2) a negative stimulation response; and (3) a non-specific artifact, are shown in Fig. 2.

An impedance change was classified as: (1) positive, stimulation-induced, and characteristic, if:

- There was a new onset of an impedance change after direct stimulation was applied;
- The signal shape of the impedance change corresponded to the stimulation-induced impedance changes of smooth muscle derived during the preclinical animal study^17^;
- The duration of the impedance change until reaching a maximum was several seconds, which correlates with a low-frequency smooth muscle contraction after spike elicitation (3–15/min)^18,19^; and
- The positive stimulation response could be verified by a negative control in surrounding tissue where no nerves were expected and stimulation led to no impedance change.

A stimulation response was classified as: (2) negative if no significant signal change could be assessed during direct stimulation of the tissue of interest (e.g., in the wound margin or adipose tissue).

Impedance changes were classified as: (3) non-specific (artifact), if:

- The impedance change could not be put into a temporal relation to the stimulation;
- The stimulation resulted in a random signal response that could be attributed to mechanical manipulation of the target organs; or
- The mentioned criteria of (1) a positive stimulation response was not fulfilled.

### Conceptional algorithm design and used data

For algorithm development and its subsequent validation, the pre-classified measurement data were divided into two data subsets. Signals from the first 15 of the 30 intraoperative cases of impedance measurement were used to develop the AMINA, while data from the other 15 cases were used to test and validate the implemented algorithm.

The first data subset included a total of 131 signal sections that were intraoperatively assessed as (1) positive stimulation-induced characteristic impedance changes. To compare signal features of the three signal classes, 131 additional signal sections with (2) negative stimulation responses and 131 signal sections with (3) artifacts were extracted from the data subset. Signal features were determined using offline signal analysis in time and time–frequency domain, with the aim of obtaining signal features for which the signals of the three classes differ significantly from each other. Based on the results of the signal pre-classification by an application specialist, it was assumed that especially (1) positive characteristic impedance changes as well as (3) artifacts can be distinguished by evaluation of the signal’s frequency components. Since the onset of the characteristic impedance change is not stationary and, thus, the frequency spectrum of the signal changes as a function of time, local characteristics of the signal in the frequency domain are of particular interest, which cannot be adequately captured by a frequency analysis, e.g., Fourier Transform (FT). For this reason, an analysis of the impedance signals in time–frequency domain using wavelet transform (WT) was applied.

**Figure 3:**
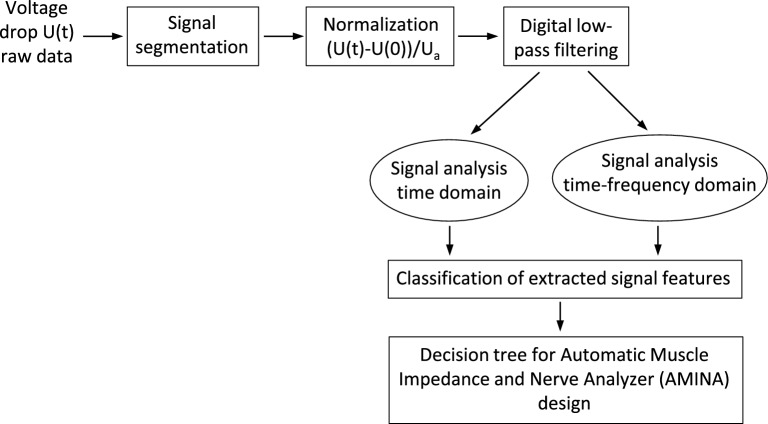
Flowchart of the algorithm design process. Segmented, normalized, and digitally filtered signal sections were used as input time series for subsequent offline signal analysis in time and time–frequency domain. Classification of the extracted signal features was used to design a decision tree underlying the Automatic muscle impedance and nerve analyzer (AMINA).

Classification of the extracted signal features was used to design a decision tree underlying the AMINA for automated, intraoperative evaluation of newly acquired impedance signals. A flowchart of the algorithm design process is shown in Fig. 3.

To avoid significant interrupts or delays of the surgeons’ workflow, it is desirable to provide intraoperative feedback to the surgeon in near-real-time. However, this requirement is in contrast to the time it takes for physiological processes of the autonomic nerve system to occur. On average, contraction of the target organs starts after 2–3 s of nerve stimulation, and the maximum contraction of smooth muscle occurs within additional 10–20 s. Therefore, no target muscle response can be expected for at least 3 s after stimulation has been applied; hence, data must be acquired over a period of > 3 s before an assessment of the stimulation response can be made. Based on these considerations, providing feedback to the surgeon 5 s after stimulation onset was assumed to be sufficient.

### Offline signal pre-processing

Signal pre-processing was applied to segment the collected data and to suppress noise or outliers in the measurement data, which could complicate automated calculation of signal parameters.

A segmented signal for offline signal analysis contained samples of the processed voltage drop U(t) over a period from stimulation onset to the recovery of the baseline impedance level, with at least the number of samples acquired during the period of stimulation. Differences in the number of samples per extracted signal section resulted from the different duration of hand-held direct nerve stimulation and the corresponding varying duration of muscle contraction. Consequently, the input signal for offline signal analysis is variable in its length.

The segmented signals were normalized to the impedance level before nerve stimulation (baseline impedance level U_a_) and digitally filtered. Since U_a_ depends on the tissue volume between the measuring electrodes and the electrical conductivity of the smooth muscle, which is proportional to its contraction state and, thus, to the resting tone of the target organ, U_a_ will be recalculated for each extracted signal section. U_a_ was defined as the mean value of the first 15 samples acquired within 1.5 s after stimulation onset to smooth out outliers in the baseline impedance level. Each segmented signal was normalized by dividing each sample by U_a_ and applying a baseline correction to zero by subtracting (U(0)/U_a_), resulting in a dimensionless discrete signal section of Y(t) = (U(t)–U(0))/U_a_.

Digital filtering was performed by low-pass filtering using a 3rd order infinite impulse response Bessel filter. A cut-off frequency of 0.15 Hz proved to be suitable for an automated calculation of signal parameters without changing relevant signal components and falsifying the result of a subsequent signal analysis. Low-pass filtering was subsequently implemented in and against the direction of time, resulting in a near-zero phase filter.

Segmented, normalized, and smoothed signal sections as well as their calculated integral and first derivative were used as input time series for the following offline signal analysis in time and time- frequency domain (see Fig. 3 for flowchart). LabVIEW 2020 (National Instruments Corporation, Austin, USA) was used for both data pre-processing and signal analysis.

### Offline signal analysis in time domain

For signal analysis in the time domain, the maximum amplitude (A_max_) within the signal waveform, the onset latency (t_0_) of the impedance change, and the maximum gradient (m) were calculated. For each signal class ((1), (2), and (3)), the arithmetic mean and median of these signal characteristics, and the distribution of the values were calculated from 131 signal sections using MATLAB® R2022b (MathWorks, Natick, MA, USA).

### Offline signal analysis in time–frequency domain

The input signal for intraoperative near-real-time signal analysis includes samples acquired during an active stimulation, which, in the case of a positive stimulation response, include a signal change up to a maximum without a subsequent relaxation phase of the muscle. Accordingly, the wavelet analysis of the signals should be designed in such a way that positive impedance changes can be detected, in particular, by their characteristic signal edge. Continuous wavelet transform (CWT) allows the use of intermediate scaling factors of the wavelet and the use of an identical time resolution of the signal in time domain and the transform coefficients for each frequency band, whereas discrete wavelet transform (DWT) provides only dyadic wavelet scaling and translation factors. Thus, the discretized CWT was believed to be more suitable for analysis of impedance signals within this work. Since the Fast Fourier Transform (FFT) of the first derivative of the impedance signal to be analyzed shows a similar frequency band as the Mexican hat wavelet, the Mexican hat wavelet was used for the application of CWT in this work.

Data analysis was performed using the LabVIEW 2020 development software (National Instruments Corporation, Austin, USA). A center frequency of the mother wavelet of F_c_ = 0.25 Hz (default in LabVIEW 2020) with a wavelet scaling factor of a = 1 and 300 scales was used, so that frequencies in the range of 2.5 Hz > F_c_ > 0.008 Hz were detectable. The translation factor k was set to 1 sample (step interval of 0.1 s at a sampling frequency of f_s_ = 10 Hz), which was found to be sufficiently precise compared with the slow physiological processes of the autonomic nervous system.

To identify the signal features relevant for software-based discrimination of the signal classes of (1) positive impedance changes and (3) artifacts, a systematic quantitative comparison of the wavelet scales at magnitude maxima of the transformation coefficients of CWT was performed using the pre-processed signal sections of the first measurement data subset. An automated detection of the magnitude maxima in the CWT scalogram including extraction of the corresponding wavelet scales was implemented in LabVIEW based on an iterative threshold-based analysis of the transformation coefficients in a sliding detection window. The distribution of the determined wavelet scales for the different signal classes was evaluated using boxplots in MATLAB® R2022b. The two-tailed t-test was used to test whether the mean wavelet scales at magnitude maxima of the two signal classes were statistically significantly different.

### Algorithm design for intraoperative (near-real-time) signal analysis

In intraoperative use, the filtered and normalized voltage drop U(t) at the urinary bladder and rectum is displayed on the neuromonitor as a function of time. When direct stimulation of a tissue portion of interest is applied by the surgeon, the corresponding impedance signal section is extracted and evaluated by the AMINA. Therefore, samples of the impedance measurement are collected in a signal buffer when direct nerve stimulation is applied. Because of the slow stimulus–response behavior of smooth muscle, a stimulation-induced response is not expected until 2–3 s after stimulation of the innervating nerves, and signal segments of at least 50 samples (5 s) are considered for intraoperative (near-real-time) evaluation. Impedance signals acquired during stimulation phases with a duration of less than 5 s are ignored and discarded in the signal buffer.

**Figure 4:**
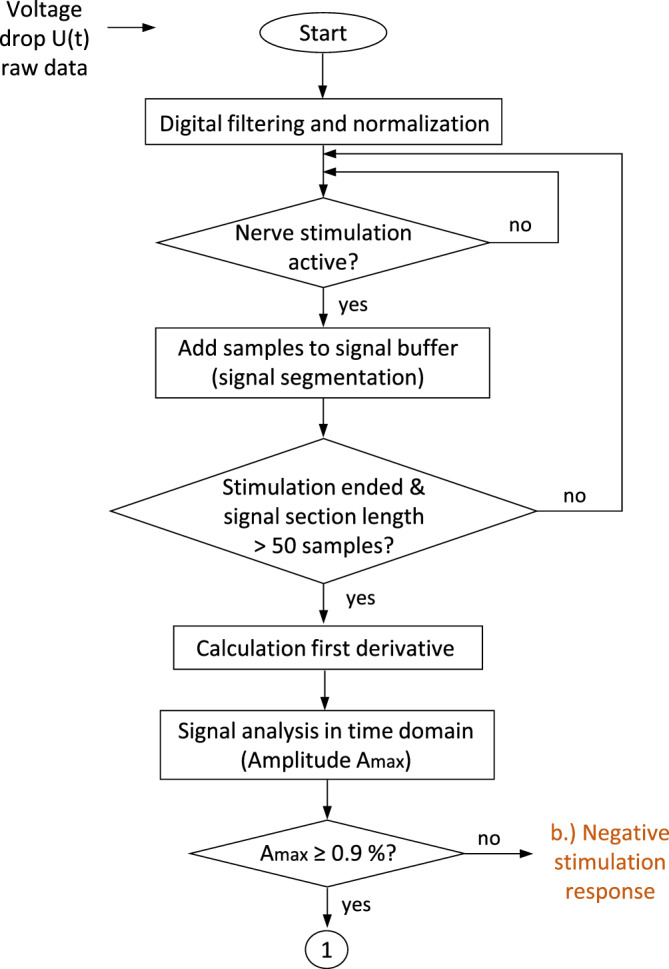
Flowchart Part I of the Automatic muscle impedance and nerve analyzer (AMINA). Pre-processed samples of the impedance signal are collected in a signal buffer during nerve stimulation. Signal analysis in time domain is performed for signal sections with > 50 samples in length. Discrimination between (2) negative stimulation responses and (1) positive or (3) non-specific responses is made based on an amplitude threshold A_max_ of 0.9%.

Pre-processed signal sections with a length of ≥ 50 samples as well as their calculated integral and first derivative are forwarded for signal analysis in the time domain including calculation of the maximum amplitude of signal change (A_max_). If A_max_ is < 0.9%, the impedance change is evaluated as not significant (see results for the offline signal analysis in time domain). In this case, the signal section is classified as (2) a negative stimulation response, based on the conclusion that no or non-functional nerves were stimulated (see flowchart in Fig. 4).

If the amplitude A_max_ is ≥ 0.9%, either a positive characteristic impedance change or a non-specific signal change (artifact) may be present. To distinguish between these two signal classes, the signal analysis in time–frequency domain is performed. The first derivative of the pre-processed signal section is transformed into the time–frequency domain using discretized CWT. The parameters of CWT are set as described above. To keep the number of calculation steps as small as possible, it was determined whether magnitude maxima are present in the CWT scalogram in the frequency band characteristic for a positive stimulation response. For this purpose, the transform coefficients in the wavelet scaling range > 37 (see results for the offline signal analysis in time–frequency domain) are extracted and subjected to magnitude maxima detection. If no extrema in this specific wavelet scaling range can be identified, the result of the signal evaluation by the AMINA is classified as (3) a non-specific impedance change (artifact), which suggests no presence of functional autonomic nerves in the field of stimulation.

If extremes characteristic of a positive stimulation response can be identified in the wavelet scaling range > 37, an additional detection of magnitude maxima in the wavelet scaling range ≤ 37 (specific for artifacts) is performed to check whether artifacts are present at similar times that may have a higher absolute magnitude and, thus, dominate signal contents. If a magnitude maximum in the frequency band of the positive impedance changes and a magnitude maximum in the frequency band of the artifacts are overlapping temporally, the absolute magnitudes of the two extrema are compared. If the magnitude of the extremum in the wavelet scaling range of the positive impedance changes is smaller, the result of the signal evaluation is (3) a non-specific artifact. If the magnitude of the extremum in the wavelet scaling range > 37 is higher, the signal is assessed as (1) a positive impedance change.

**Figure 5:**
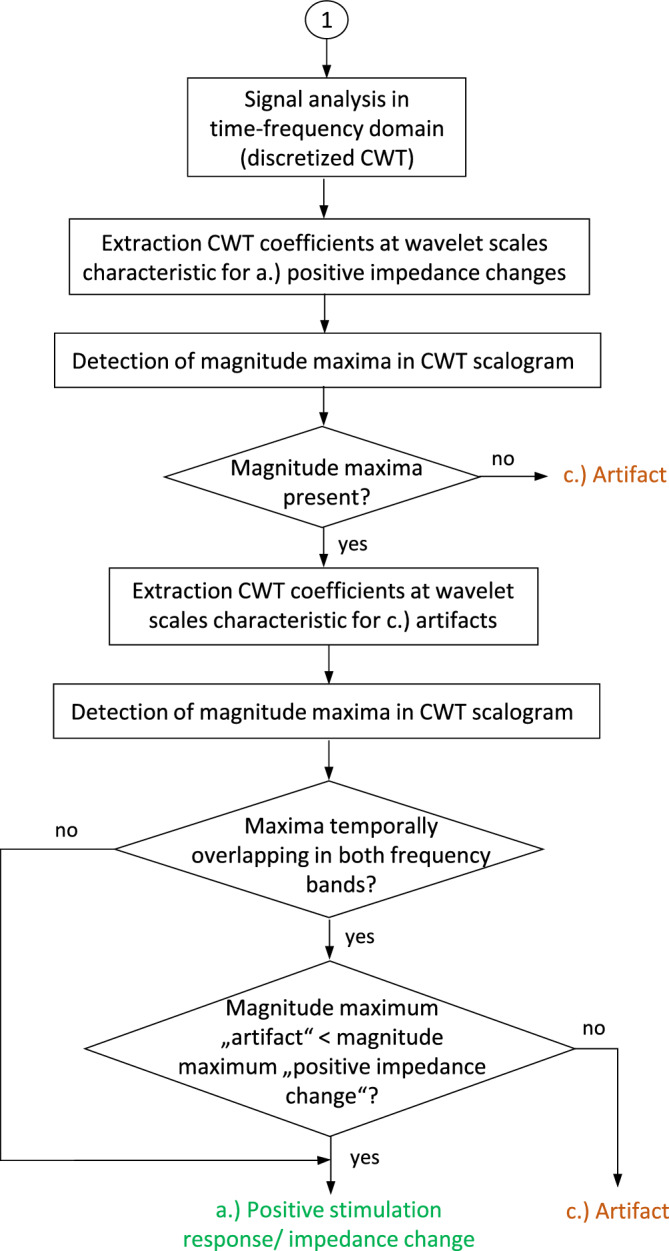
Flowchart Part II of the Automatic muscle impedance and nerve analyzer (AMINA). Signal sections, which are assessed as (1) positive or (3) non-specific stimulation responses are forwarded to signal analysis in the time–frequency domain using discretized CWT. The distinction between the two signal classes is based on the wavelet scale associated with the magnitude maximum of the transformation coefficients of the CWT.

If there is no temporal overlap of an artifact with a positive impedance change, it can be directly concluded that the examined signal section contains (1) a positive characteristic impedance change related to a localization of functional autonomic nerves in the field of stimulation. A flowchart of the Automatic muscle impedance and nerve analyzer (AMINA) is depicted in Fig. 5.

### Algorithm validation

From the second data subset (the second 15 cases of the clinical investigation considered for validation), 108 signal sections were intraoperatively pre-classified as positive characteristic impedance changes. To evaluate the implemented signal assessment by the AMINA for all three signal classes, an identical number of manually pre-classified signal sections with negative stimulation responses and non-specific impedance changes (artifacts) was extracted from the raw data of the data subset. Thus, for each of the three signal classes, previously unanalyzed data were included in algorithm validation.

The intraoperative (near-real-time) application of the AMINA was simulated by streaming the measured data. The signal evaluation results obtained by the AMINA were stored in data arrays, with traceability to the underlying pre-classification results of the application specialist, which was used to determine the sensitivity and specificity of the proposed method. The sensitivity P_1_ was calculated as the number n_1_ of (1) positive impedance changes, which were correctly assessed: P_1_ = n_1 _* 100%/108.

The specificity P_2_ of the proposed method was determined by the number n_2_ of (2) negative stimulation responses and n_3_ of (3) artifacts, which were correctly detected by the AMINA as (2) or (3) from the total number of negative stimulation responses and artifacts: P_2_ = (n_2_ + n_3_) * 100%/216.

To determine whether the distribution of the signal features was similar between the two data subsets for algorithm design and testing, the arithmetic mean value, the standard deviation, and the parameter ranges (excluding 5% outliers) of A_max_ were calculated for (1) positive responses from both data subsets.

The runtime of the algorithm was determined in the development environment (non-compiled application) of LabVIEW 2020 using a Lenovo Thinkpad E14 Gen 2 with an 11th Gen Intel Core i7-1165G7 2.8 GHz processor, 16 GB RAM, and a 64-bit Windows 10 operating system. The runtime of the signal evaluation procedure was calculated during the application to two impedance signals simultaneously (in the intraoperative application corresponding to a two-channel impedance measurement on the urinary bladder and rectum), using the timer function in LabVIEW 2020. Since with a higher number of samples per signal section an increasing time requirement for the transformation of the signals into the time–frequency domain was to be expected, the runtime was determined for signal sections with different lengths of 50, 75, 100, 125, 150, 175 and 200 samples in 10 runs each. By means of polynomial regression, a function for the determination of the average runtime depending on the number of samples was determined.

## Results

**Figure 6:**
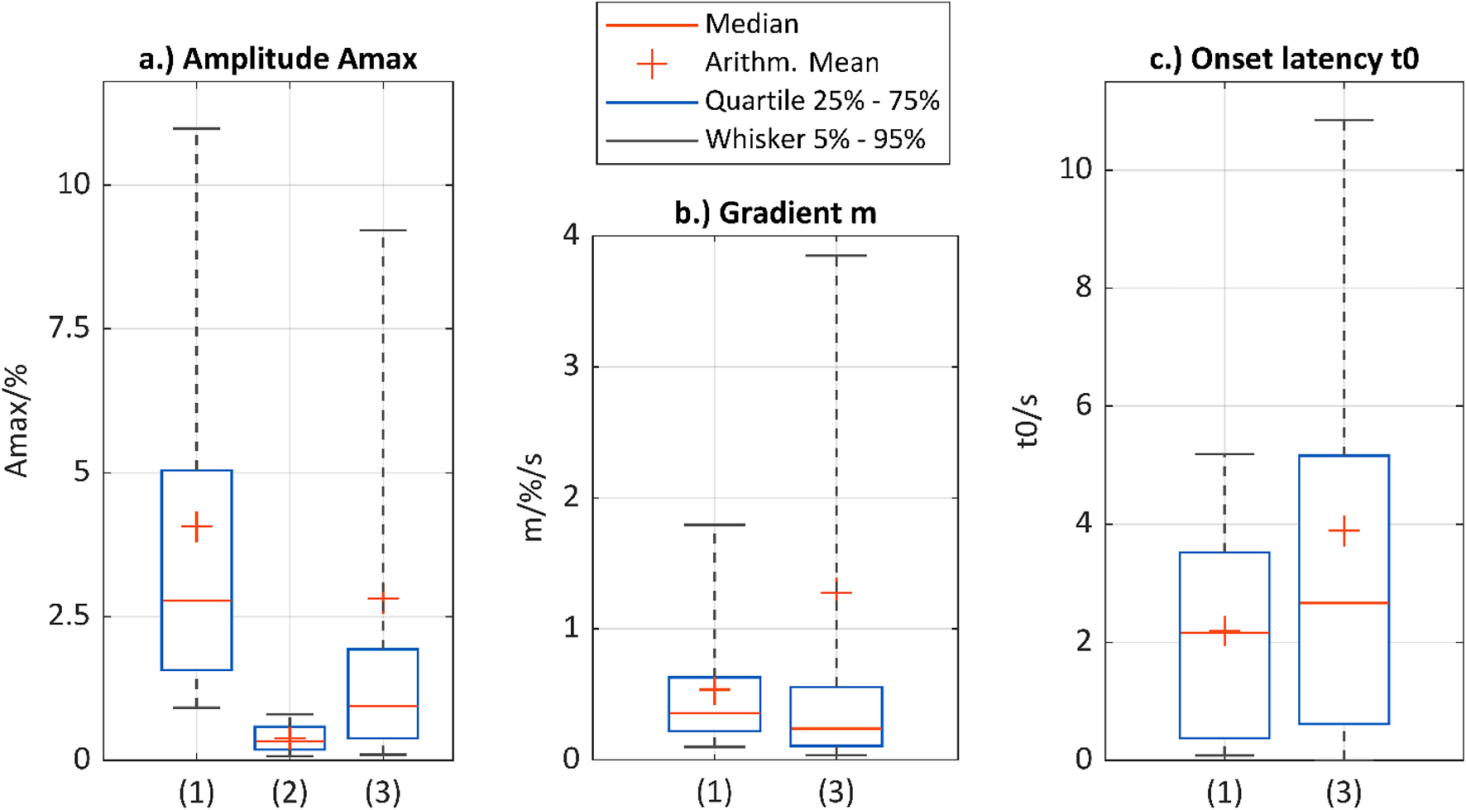
Boxplot of amplitude Amax (**a**), gradient m (**b**) and onset latency t_0_ (**c**) for comparison of (1) positive impedance changes, (2) negative stimulation responses, and (3) artifacts (n = 131 per signal class from case 1 to 15 of the clinical investigation/patients LB-01–LB-16).

Figure 6 shows the results of the calculated signal parameters A_max_, t_0_ and m in the time domain for (1) positive impedance changes, (2) negative stimulation responses, and (3) artifacts. The value ranges of the three signal parameters in the time domain for (1) positive impedance changes are not significantly different from those of (3) artifacts. The mean values of the onset latency and the maximum gradient for artifacts (t_0_ = 3.9 s and m = 1.3%/s) are higher than for positive impedance changes (t_0_ = 2.2 s and m = 0.5%/s). However, since the signal parameters for positive impedance changes are within the parameter range of artifacts, the two signal classes cannot be distinguished on the basis of these signal parameters in the time domain. A supplementary statistical analysis of the comparison results was therefore not used.

From the comparison of (1) positive impedance changes with (2) negative stimulation responses, it is evident that signals of these categories can be distinguished based on A_max_. Impedance changes classified intraoperatively as characteristic of a stimulation-induced contraction have an amplitude of ≥ 0.9% (exclusion of 5% outliers, lower whisker). In contrast, signal fluctuations in signal sections with negatively rated stimulation responses have amplitudes of ≤ 0.8% (exclusion of 5% outliers, upper whisker). A paired two-sided t-test was used to additionally test whether the mean maximum amplitude A_max_ with µ_1_ = 4.1% and standard deviation sd = 3.7% of positive impedance changes is significantly different from the mean A_max_ of impedance changes in negative stimulation responses with µ_2_ = 0.4%:

$$\begin{aligned} & Null\,\,hypothesis\,H0 = \mu 1 - \mu 2 = 0 \\ & Alternative\,\,hypothesis\,H1 = \mu 1 - \mu 2 \ne 0 \\ \end{aligned}$$

$$T=\frac{\mu 1-\mu 2}{sd*\frac{1}{\sqrt{n}}}= \frac{\left(4.1-0.4\right)\%}{3.7 \%*\frac{1}{\sqrt{131}}}= 11.45$$

$$t\left(1-\frac{\alpha }{2}, n-1\right)=1.978$$

$$H0\,rejection\,\left(-\infty ,-t\right] \bigcup [t,\infty )$$

with T = test statistic, t = test value, and sd = standard deviation. As $\infty >T>t$, the null hypothesis H0 can be rejected. Thus, the maximum amplitudes are significantly different for positive and negative signal responses, which can be used to delineate the two signal classes within the AMINA algorithm. As a consequence, the analysis of the negative stimulation responses in the time–frequency domain is not necessary.

**Figure 7:**
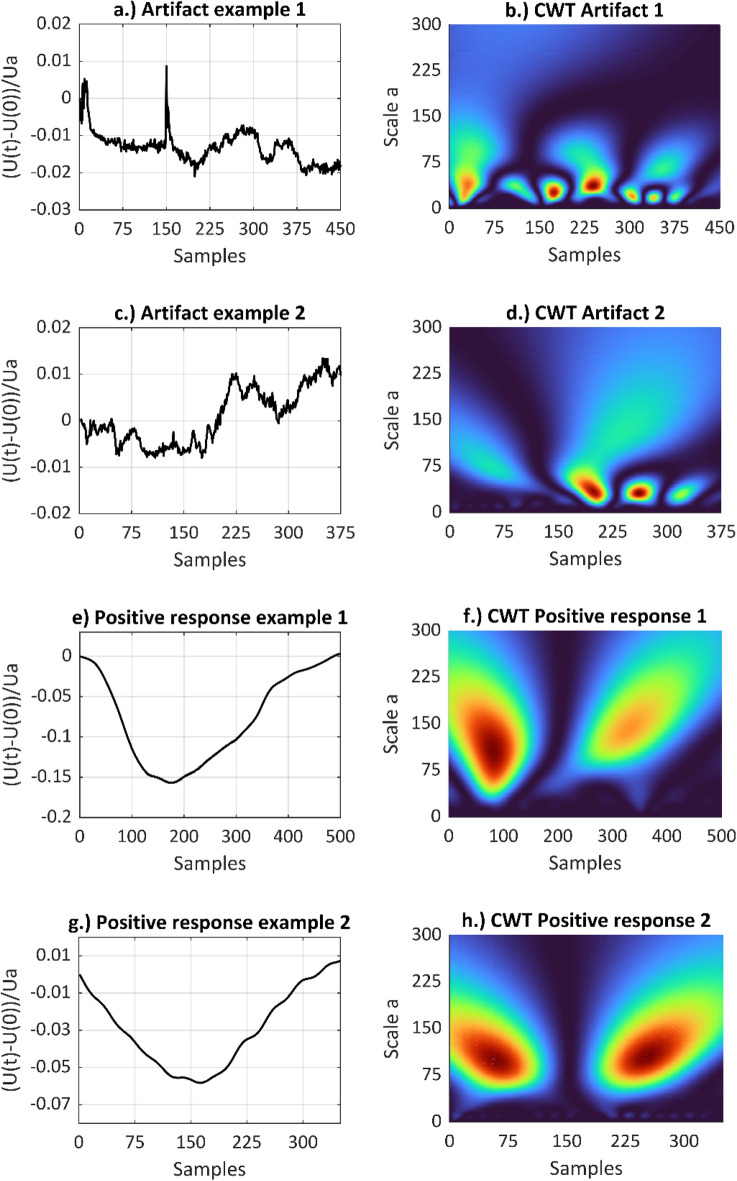
Signal sections with artifacts in the time domain (top left, **a** and **c**), and corresponding CWT scalograms (top right, **b** and **d**), signal examples from case 3 of the clinical investigation/patient LB-04), and signal sections with positive stimulation responses in the time domain (bottom left, **e** and **g**) and corresponding CWT scalograms (bottom right, **f** and **h**), signal examples from case 1 and case 15 of the clinical investigation/patient LB-01 and LB-16). The examples show clear differences in the wavelet scale at maximum magnitudes of the CWT transformation coefficients.

Figure 7 shows the results of the CWT. Specifically, two signal sections of positive impedance changes and two signal sections with artifacts are shown both in time (left) and time–frequency domain (right). The examples show clear differences in the wavelet scale at maximum magnitudes of the CWT transformation coefficients, with a wavelet scale > 75 for positive signal responses and a wavelet scale <  < 75 for artifacts. Different frequency components in a signal section with positive impedance changes and artifacts lead to differences in the wavelet scaling at maximum coefficients, thus, allowing to distinguish between the two signal classes.

**Figure 8:**
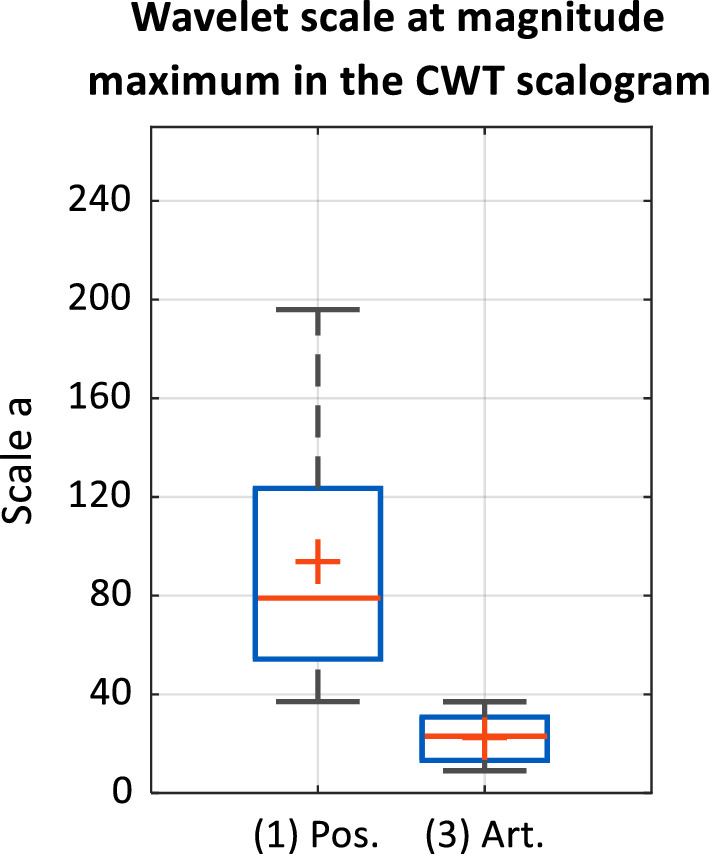
Boxplots of wavelet scales at maximum magnitudes of transformation coefficients of CWT of (1) positive characteristic impedance changes and (3) artifacts; n = 131 per signal class from cases 1 to 15 of clinical investigation/patient LB-01–LB-16).

The result of the systematic evaluation of the wavelet scales at magnitude maxima in the CWT scalogram of the 131 positively pre-classified impedance changes of the clinical investigation (first data subset) compared with an identical number of artifacts is shown in Fig. 8. With a mean wavelet scale of 94, there is a maximum similarity of the wavelet and the first derivative of the impedance signal. The arithmetic mean of the wavelet scale at which the first derivative of a signal section with non-specific impedance changes (artifacts) shows maximum similarity with the wavelet is 23.

The signal edge of positively classified impedance changes varies due to differences in the contraction behavior of the smooth muscle during repeated stimulations. Therefore, frequency components of the signal sections are subject to scatter in the time–frequency domain. If 5% of the values at the upper and lower limits of the value range of the determined wavelet scales are excluded as outliers, the wavelet scales lie in the range of 37–196 for magnitude maxima in the scalogram of the positive impedance changes, and in the range of 9–37 for artifacts. The two signal classes can, thus, be delimited based on the criterion of a wavelet scale of 37 at the magnitude maximum of the transformation coefficients.

A paired two-tailed t-test was used to additionally test whether the mean wavelet scale µ_1_ = 94 with the standard deviation sd = 49 resulting from the analysis of positive impedance changes was significantly different from the mean wavelet scale µ_2_ = 23 from the analysis of artifacts:

$$Null\,\,hypothesis\,H0= \mu 1-\mu 2=0 Alternative\,\,hypothesis\,H1= \mu 1-\mu 2\ne 0$$

$$T=\frac{\mu 1-\mu 2}{sd*\frac{1}{\sqrt{n}}}= \frac{\left(94-23\right)\%}{49*\frac{1}{\sqrt{131}}}= 16.6$$

$$t\left(1-\frac{\alpha }{2}, n-1\right)=1.978$$

$$H0\,rejection\,\left(-\infty ,-t\right] \bigcup [t,\infty )$$

As $\infty >T>t$, the null hypothesis H0 can be rejected. Thus, the mean values of the wavelet scales of the positive impedance changes and the artifacts are statistically significantly different. Accordingly, this approach can be used by AMINA as a criterion to distinguish positive responses from artifacts.

Of the 108 signal sections of the second data subset pre-classified as positive impedance changes, 104 were judged as such by the AMINA. Of the four signals falsely classified as negative, three were assessed as artifacts and one signal section as a negative stimulation response. The sensitivity P_1_ of the method was, thus, 96.3%. Of the signal segments with negative stimulation responses, three were classified as false positives, 103 correctly as (2) negative stimulation responses, and one signal segment as (3) artifact. Signal assessment of (3) the artifacts revealed 16 of the 108 signals were assessed as false positives and 92 signals correctly identified by the AMINA as (2) negative or (3) artifact. The specificity P_2_ of the method was, thus, 91.2%.

The determination of the comparability of the two data subsets for algorithm design and testing showed similar distributions of the signal features of positive impedance changes. A_max_ for the first data subset resulted in a mean value of the maximum amplitude A_max_ of µ = 4.1%, a standard deviation of std = 3.7% and a parameter range of A_max_ = 0.9% to 10.9% (exclusion of 5% outliers, upper and lower whisker). A_max_ for the second data subset used for algorithm testing resulted in a mean value of the maximum amplitude A_max_ of µ = 4.2%, a standard deviation of std = 3.3% and a parameter range of A_max_ = 0.8% to 10.1% (exclusion of 5% outliers, upper and lower whisker).

The determined runtime of the AMINA shows a non-linear increase with an increasing number of samples of the input signal. The regression function determined on the basis of the mean values of the 10 measurement runs is y = 0.0006052 * x^3^− 0.04135 * x^2^ + 5.4006  * x− 185. Assuming an average duration of direct nerve stimulation of 10 s (100 samples), the mean duration of signal evaluation by the AMINA for a two-channel impedance measurement is 582 ms. The requirement for a signal assessment duration of 3–5 s, within which feedback should be given to the surgeon, is therefore fulfilled. If the accepted duration of the signal assessment is 3 s maximum, the input signal must not contain more than 180 data points. Since the onset of the impedance change to the maximum impedance change occurs on average in a time range of 2.2–10.0 s (22–100 samples) after direct nerve stimulation onset (see results of the offline signal analysis in time domain), an input signal of a maximum length of 180 samples is sufficient for reliable signal assessment.

## Discussion

The results of the signal analysis in the time domain show that characteristic impedance changes caused by smooth muscle contraction cannot be significantly distinguished from artifacts based on calculated signal parameters in the time domain. The signal shape of impedance changes resulting from mechanical manipulation in the surgical field can vary depending on the specific movements induced at the target organ, making them random in nature. These impedance changes occur due to external influences rather than stimulation, which means that the onset latency t_0_, for instance, cannot serve as a characteristic for the signal class. Furthermore, the amplitude of the signal change in artifacts depends on the extent of mechanical manipulation acting on the target organ and is, thus, also random. Independent of the individual, it can therefore be expected that calculated signal parameters of artifacts such as A_max_ or t_0_ can take on values similar to those of characteristic impedance changes.

The results of the signal analysis in the time–frequency domain show that contraction-related positive impedance changes can be distinguished from artifacts using calculated signal parameters in the time–frequency domain. The signal parameters relevant for the discrimination of signals were successfully identified by CWT. The determined wavelet scales at maximum magnitudes of the transformation coefficients of the two signal classes were statistically significantly different. The implementation of a decision tree for the AMINA algorithm for intraoperative application was, thus, possible.

Scientific work from relevant literature on the classification of biosignals for similar applications utilizes, for example, time–frequency analysis of EMG for identification and characterization of patient-specific muscle movements to develop algorithms for early detection of neuromuscular diseases^20,21^ or to control motorized prostheses^22,23^. Boyer et al. published 2021 their investigation of EMG obtained with surface electrodes. To allow pattern recognition, previously defined muscle activities were established, which were elicited by awake study participants. The median frequency of the spectral power density (PSD) determined by short-time Fourier transform (STFT) or CWT was thereby defined as an indicator of muscular fatigue. The wavelet transformation of the EMG signals was thereby performed for pre-processed segmented EMG data using the Morse wavelet. With respect to the present work, the median frequency is not considered to be sufficiently precise to detect the frequency of an onset characteristic impedance change and to distinguish it from the frequency of an artifact. In the signal detection implemented in this work, it is crucial to determine the frequency of the impedance signal at a specific time. In contrast, in the work of Boyer et al., an averaged median frequency was calculated that results from a signal spectrum over a longer duration.

A similar analysis to the work of Boyer et al. was published by Hussain et al. in 2012, in which DWT using the Haar, Daubechies, or Symlet wavelets was applied to decompose EMG activity to identify muscle fatigue. In addition to determining the median frequency of the power spectrum, a higher-order spectrum analysis (bispectrum) was used. Based on the results of the linearity test or the test of the normal distribution of the squared normalized version of the bispectrum (bicoherence), muscle fatigue was detected. The lower the median frequency or the less normally distributed the bicoherence, the larger the decrease in muscle tone and, thus, muscle fatigue was inferred^21^. In their analysis of EMG in 2014, Wang et al. determined the correlation dimension of determined coefficients of DWT. The resulting clustering of transformation coefficients was used to delineate different motor movement patterns^22^.

The only application of time–frequency analysis of intraoperative stimulated signals to date was published by Hu et al. in 2003. Time-related changes of the frequency spectrum of continuously derived sensory evoked potentials (SEP) compared to baseline are considered as an indicator of impending impairments of afferent pathways of the spinal cord during interventions for the correction of scoliosis. Signal sections of averaged SEP signals were transformed to the time–frequency domain by STFT using the Hanning window. Changes in the magnitude maximum in the resulting power spectrum with determination of the corresponding time and frequency were compared with changes in the amplitude and latency of the SEP signals in the time domain. The time–frequency analysis of the signals is considered more stable with respect to signal changes that were not associated with neurological deficits, suggesting better specificity of the signal analysis method. The lack of time resolution, known for STFT, was found to be limiting^24^. This work successfully implements time–frequency analysis for the assessment of signals in intraoperative neuromonitoring. The reason for the currently still missing transfer into clinical routine is assumed to be the fuzziness of time resolution. Further research to optimize the analysis would therefore be needed.

During nerve localization in the surgical area with a hand-held stimulation probe, a high sensitivity of the method is preferable to a high specificity. For example, if the result of the signal assessment is a (2) negative stimulation response or a (3) artifact, the surgeon will assume that there are no nerves in the area of the stimulation probe. Consequently, the surgeon is not encouraged to explicitly preserve nerves; consequently, dissection and, if necessary, tissue resection in this area can continue unimpeded. If the stimulation response is falsely assessed as negative by the software-based procedure, there is a risk of unintentional damage to nerves, resulting in postoperative neurological sequelae for the patient. In the case of a positive signal assessment, on the other hand, the surgeon assumes the presence of autonomous nerves in the area of the stimulation probe and, therefore, further dissection is carried out in a nerve-sparing manner. In the case of a false positive signal interpretation, the surgeon is incorrectly encouraged to preserve tissue, which is more time-consuming, but not associated with an increased risk of nerve damage. To improve the 91.2% specificity of the proposed method, the criteria for discriminating signals by the AMINA would need to be adjusted so that fewer artifacts are falsely judged as positive impedance changes (increase threshold wavelet scale). This would cause more positive impedance changes to be judged as artifacts, thus, reducing sensitivity. Since a high sensitivity is more important than a high specificity, the results of the validation achieved so far can be rated as satisfactory.

The clinical investigation underlying this work was performed to investigate the feasibility of bioimpedance measurement as a new neuromonitoring method. Due to the monocentric study design with a sample size of 30 patients only, the study lacks a representative proof of long-term clinical benefit, as well as an evaluation of influences by different principles of use due to different surgeons and neuromonitoring users and the assessment of influences by the patient population (such as gender, body weight, pre-treatments, etc.). To collect representative data, multicenter studies including higher samples sizes are needed. To further improve signal assessment by the AMINA, collection of data from a representative sample size is necessary. Factors that may influence signal characteristics are the intensity of the stimulation current reaching the nerves, the exact position of the recording electrodes on the target organ as well as the individual anatomy of a patient (amount of visceral fat or differences in the course of innervation). If an exposed main branch of the pelvic nerve plexus (superior hypogastric plexus or inferior right/left plexus) is stimulated, a stronger stimulation response can be expected than if only individual fine branches of the plexus are reached. A systematic analysis of these influences can only be performed after collection and analysis of a larger sample of data. It could additionally evaluate whether signal characteristics may be patient-specific. In this case, the sensitivity and specificity of software-based signal evaluation can be improved by developing a learning algorithm that analyzes the signals acquired at the beginning of a surgery and determines and optimizes threshold values for classifying the signals based on that algorithm. Furthermore, the availability of comprehensive measurement data from the practical application of impedance measurement as a neuromonitoring method allows the exploration of alternative implementations of the AMINA. The realization of different procedures for software-assisted signal assessment and a systematic comparison of the results obtained in this process was not considered to be purposeful in the context of this work, since a possible variability of the signals and their influence on the sensitivity and specificity of the procedure cannot yet be adequately evaluated due to the limited data available.

The currently only available alternative method for intraoperative pelvic neuromonitoring (EMG and bladder manometry) uses a threshold-based signal evaluation. Changes in the bladder pressure signal during stimulation of autonomic nerves are determined relative to a baseline level and assessed as stimulation-induced above a threshold of 1 cm H_2_O. To enhance signal interpretation for the surgeon, random signal changes that are not related to a stimulation response and may cause incorrect signal assessment are suppressed by smoothening the pressure signal. For this purpose, superimposed artifacts resulting from lifting and lowering the abdominal wall due to the patient's mechanical ventilation are reduced using a sample-and-hold circuit and digital filtering by a finite impulse response filter. Moreover, signal drifts are eliminated by software-based baseline correction of the pressure signal^25^. Nevertheless, using threshold-based methods by amplitude and/or latency calculations, artifacts with similar signal parameters cannot be distinguished from stimulation-induced muscle responses, which can lead to false positive results of signal evaluation.

## Conclusion

The method researched in this work represents a first promising implementation of software-based signal assessment, which can be improved on the basis of further measurement data. Differentiation between stimulation-induced positive impedance changes, negative responses and artifacts for intraoperative pelvic neuromonitoring can be accomplished by calculating signal characteristics in the time and time–frequency domain, which facilitates and automates signal interpretation for the surgeon.

## Data Availability

All data generated or analysed during this study are included in this published article.
